# Multiple mucoceles of the lower lip: A case report

**DOI:** 10.1002/ccr3.2253

**Published:** 2019-06-07

**Authors:** Atsushi Abe, Kenichi Kurita, Hiroki Hayashi, Masashi Minagawa

**Affiliations:** ^1^ Department of Oral and Maxillofacial Surgery Nagoya Ekisai Hospital Nagoya Japan; ^2^ Department of Oral and Maxillofacial Surgery Aichi‐gakuin University Nagoya Japan

**Keywords:** lower lip, mucocele, multiple

## Abstract

A 2‐year‐old girl developed three mucoceles on lower lip, probably due to her habit of rolling lower lip behind the maxillary anterior teeth. The spacing in the dentition and mechanical irritation from habitual lip biting may have caused the mucoceles. At 3 years after excision surgery, the patient remained recurrence‐free.

## 1 INTRODUCTION

A mucocele is caused by impaired saliva flow from a salivary gland and is the most common type of cyst that occurs in oral soft tissues. Most mucoceles develop as a solitary lesion, and only a few cases of multiple lesions have been reported.^1^ We herein report our experience with a case of multiple mucous cysts in a 2‐year‐old girl.

## 2 CASE REPORT

**Figure 1:**
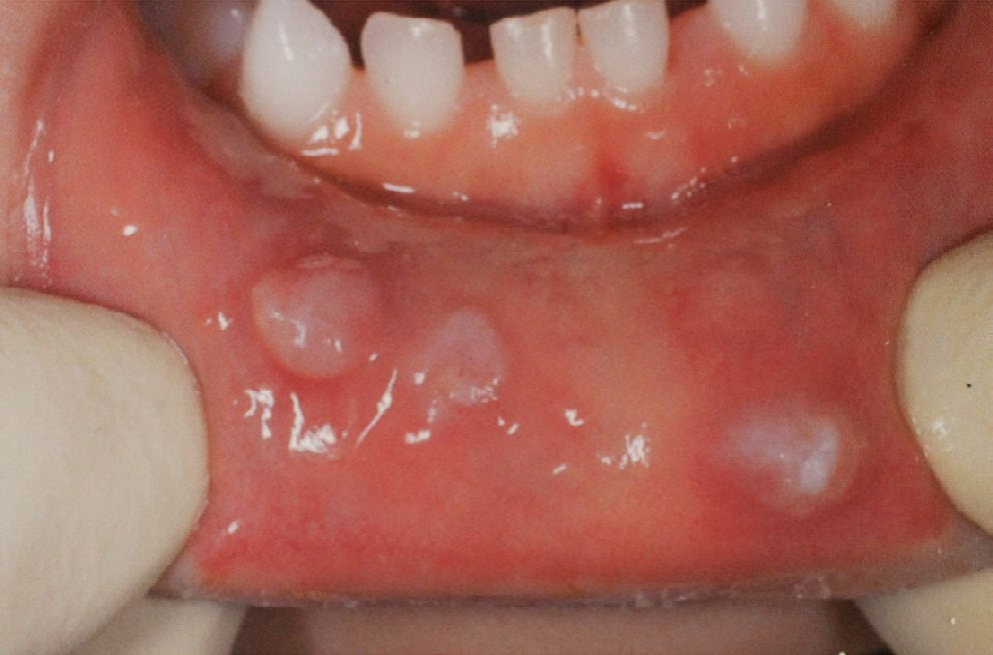
Multiple mucoceles on the lower lip. There are three well‐circumscribed masses on the lower lip, corresponding to the locations of the spaces between the primary teeth

**Figure 2:**
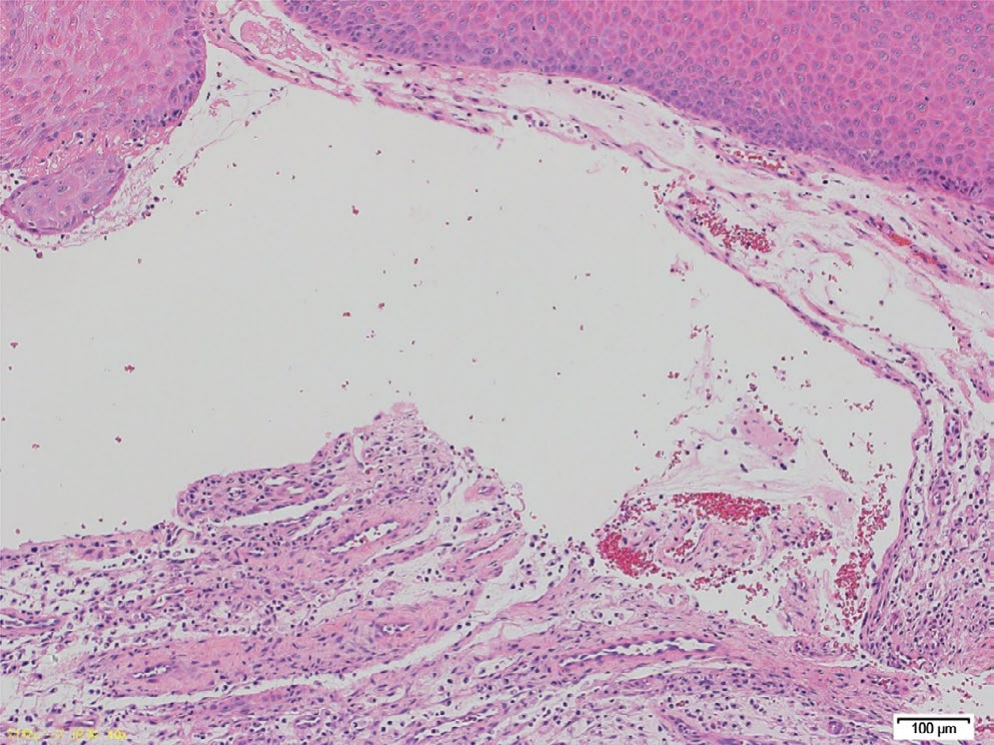
Histopathologic features of mucoceles. The cystic cavity is surrounded by granulation tissues and fibrous connective tissues, and contains mucosubstances and foam cells. Hematoxylin–eosin stain ×100

Our patient was a 2‐year‐old female infant. She presented with three painless vesicles on the lower lip that had emerged 3 weeks earlier. The vesicles had been left untreated due to their fluctuation in size. However, a local dentist who saw the patient for a routine checkup recommended a thorough examination of the vesicles, and thus referred her to our hospital. Her medical and family histories were unremarkable. At the initial visit, three well‐circumscribed round masses, each measuring approximately 5 mm in diameter, were found on the lower lip. The lesions were bluish fluctuant masses covered with normal mucosa. No pain or congestion was noted. The facial appearance was symmetric without any skin rashes. No swelling or tenderness was present in the submandibular or cervical lymph nodes. The patient had no fever and her food intake was good. The differential diagnosis of mass lesions in lower lip is salivary gland tumors, fibroma, and hemangioma. Salivary gland tumors of the minor glands can occur on the lip but are more commonly seen in the upper lip rather than in the lower lip. Fibroma is commonly pink and nonfluctunt. Hemangioma can undergo transient reduction in size under pressure. Based on these findings, the lesions were diagnosed as multiple mucoceles of the lower lip. The mucoceles were individually excised, along with the surrounding minor salivary glands, under local anesthesia. We removed three spherical masses, each measuring 5 × 5 × 5 mm in size (Figure 1). Histopathological analysis revealed that the cysts were present within connective tissues and lacked lining epithelium. In the minor salivary gland tissues, lymphocytic infiltration was noted around the ducts. These histopathological findings led to a diagnosis of mucoceles (Figure 2).

Her postoperative course was uneventful without any infection or wound dehiscence. To date, 3 years after the operation, the patient has not experienced any recurrence. Informed consent was obtained from the patient's parents, and the procedures were performed in accordance with the Helsinki Declaration.

## 3 DISCUSSION

A mucoceles is the most common type of cyst that occurs in oral soft tissues. Chi et al^1^ reported a large‐scale case series involving 1824 patients with mucoceles, and revealed that the most common site for mucoceles was the lower lip (81.9%), followed by the floor of the mouth (5.8%), and the ventral surface of the tongue (5.0%). The reason for the proclivity of mucoceles to occur on the lower lip is unclear, but several studies have reported possible reasons, which are associated with parafunctional habits (such as lip biting),^2^ differences in the mobility of the upper and lower lips,^3^ or differences in salivary gland density.^4^, ^5^ Additionally, most mucoceles develop as a solitary lesion. Multiple mucoceles occur infrequently, and the incidence rate thereof is reported to be approximately 2%.^1^, ^6^ Because of this low incidence, it is unclear whether the development of multiple mucoceles is related to patient age and the site of the lesion.

The most likely causes of mucous cysts are injuries or chronic stimulation.^1^, ^7^, ^8^ In the present case, the patient had a habit of biting the lower lip behind the maxillary anterior teeth, and the presence of many spaces between the primary teeth caused the mesial and distal corners of the teeth to come into contact with the lip, presumably resulting in chronic stimulation, which led to the development of multiple mucoceles. The habitual lip biting that put lip into interdental space may case multiple mucoceles. Therefore, it is necessary to try the improvement of the bad habit before removing surgically. In this case, we should have tried the improvement of the bad habit. However, this patient is a 2‐year‐old girl and is too young to be able to understand the necessity of improving the bad habit. Therefore, we gave priority to surgical resection.

Histopathologically, mucoceles can be classified into (a) the extravasation type (without epithelial lining of the cyst wall) and (b) the retention type (with epithelial lining of the cyst wall).^9^ The incidence of mucous retention cysts is low, and most mucoceles are of the extravasation type. In the present case, all three lesions lacked epithelial lining and were therefore diagnosed as mucous extravasation cysts.

Mucoceles are located in submucosa and appeared as translucent small blisters covered with normal mucosa. Mucoceles can rupture due to friction from eating, and they may disappear, but then often recur. Therefore, the optimal treatment is to excise the lesions along with the surrounding minor salivary glands. As alternative treatment options, cryotherapy^10^ and local steroid therapy^11^ have been reported. However, excision is known to yield the most favorable outcomes.

When performing excision in a patient with multiple mucoceles, as in the present case, potential lip deformities caused by suturing is a concern. However, in the present case, each cyst was small and spatially separated from one another. Therefore, the lesions were removed separately, and the wounds individually sutured, which resulted in an uneventful postoperative course without lip deformities or recurrence. The recurrence rate of mucoceles after excision is approximately 7%; many of these are of the extravasation type, as in the present case. We have continued to follow up the patient while providing guidance for improving the parafunctional habit linked to the development of the mucoceles. The clinical course of the patient has been favorable without any recurrence at the 3‐year follow‐up.

## 4 CONCLUSION

We reported a case of multiple mucoceles on the lower lip in a 2‐year‐old girl. The reason for the occurrence of multiple cysts on the lower lip in this case may have included the habit of lip biting, spaces between the primary teeth, and the density of the minor salivary glands in the lower lip.

## CONFLICT OF INTEREST

None declared.

## AUTHOR CONTRIBUTION

AA: developed the concept and design of the study. KK: revised critically the manuscript for important intellectual content and gave the final approval of the version to be submitted. HH and MM: drafted the article.
